# High-Throughput Oxford Nanopore Sequencing Unveils Complex Viral Population in Kansas Wheat: Implications for Sustainable Virus Management

**DOI:** 10.3390/v17010126

**Published:** 2025-01-17

**Authors:** Nar B. Ranabhat, John P. Fellers, Myron A. Bruce, Jessica L. Shoup Rupp

**Affiliations:** 1 Department of Plant Pathology, Throckmorton Plant Science Center, Kansas State University, Manhattan, KS 66506, USA; nranabhat@utk.edu (N.B.R.); myronbruce@ksu.edu (M.A.B.); 2 Hard Winter Wheat Genetics Research Unit, United States Department of Agriculture-Agricultural Research Service (USDA-ARS), Manhattan, KS 66506, USA; john.fellers@usda.gov

**Keywords:** wheat virus, virome, RNA viruses, phylogeny, mixed infection, host plant resistance, co-infection, virus population

## Abstract

Wheat viruses are major yield-reducing factors, with mixed infections causing substantial economic losses. Determining field virus populations is crucial for effective management and developing virus-resistant cultivars. This study utilized the high-throughput Oxford Nanopore sequencing technique (ONT) to characterize wheat viral populations in major wheat-growing counties of Kansas from 2019 to 2021. Wheat leaves exhibiting virus-like symptoms were collected, total RNA was extracted, and cDNA libraries were prepared using a PCR-cDNA barcoding kit, then loaded onto ONT MinION flow cells. Sequencing reads aligned with cereal virus references identified eight wheat virus species. *Tritimovirus tritici* (wheat streak mosaic virus, WSMV), *Poacevirus tritici* (Triticum mosaic virus, *TriMV*), *Bromovirus BMV* (brome mosaic virus, *BMV*), as well as *Emaravirus tritici*, *Luteovirus pavhordei*, *L. sgvhordei*, *Bymovirus tritici*, and *Furovirus tritici.* Mixed infections involving two to five viruses in a single sample were common, with the most prevalent being WSMV + TriMV at 16.7% and WSMV + TriMV + BMV at 11.9%. Phylogenetic analysis revealed a wide distribution of WSMV isolates, including European and recombinant variants. A phylogenetic analysis of *Emaravirus tritici* based on RNA 3A and 3B segments and whole-genome characterization of *Furovirus tritici* were also conducted. These findings advance understanding of genetic variability, phylogenetics, and viral co-infections, supporting the development of sustainable management practices through host genetic resistance.

## 1. Introduction

Kansas is one of the top wheat-producing states in the United States. Kansas ranked second in 2020 and first in 2021 in the United States having produced 7.654 million tons and 9.906 million tons, respectively (USDA, National Agricultural Statistics Service, 2019 and 2020). Despite its prominence in wheat production, the state faces challenges from various biotic agents including fungi, bacteria, viruses, insects, and nematodes. The estimated history of cumulative disease loss for Kansas wheat from 1976 to 2020 ranged from 0.2% to 22.2% [1]. In 2020 and 2021, the cumulative loss of wheat by disease (excluding nematodes) was estimated to be 10.8% and 16.2% or about 31.8 million bushels and 70.4 million bushels, respectively [1,2].

Viral pathogens pose a significant threat to wheat production and have both qualitative and quantitative impacts on wheat production [3,4,5,6]. Wheat viruses are transmitted by vectors such as aphids, mites, plasmodiophorid *Polymyxa graminis*, or leafhoppers [7]. In Kansas, the prevalent wheat viral diseases are barley yellow dwarf, the wheat streak mosaic (WSM) complex, and soilborne wheat mosaic. Barley yellow dwarf disease is caused by the infection of several species of barley yellow dwarf viruses (BYDVs), including *Luteovirus pavhordei* (BYDV-PAV), *L. pashordei* (BYDV_PAS), and *L. mavhordei* (BYDV-MAV) (Virus Taxonomy 2023 Release), each vectored by a different aphid species. WSM disease can be caused by three distinct viral pathogens, including *Tritimovirus tritici* (wheat streak mosaic virus, WSMV), *Poacevirus tritici* (Triticum mosaic virus, TriMV), and *Emaravirus tritici* (High Plains wheat mosaic emaravirus, HPWMoV). All three viruses are transmitted by wheat curl mite (*Aceria tosichella* Keifer). Viruses transmitted by *Polymyxa graminis*, *Bymovirus tritici* (wheat spindle streak mosaic virus, WSSMV), and *Furovirus tritici* (soilborne wheat mosaic virus, SBWMV) are found in the soil and, though less common, are detected routinely each year. *Luteovirus sgvhordei* (cereal yellow dwarf virus (CYDV)) and *Bymovirus triticitessellati* (wheat yellow mosaic virus) are occasionally found [8,9,10]. Using next-generation sequencing, *Bromovirus BMV* (brome mosaic virus, BMV) and cocksfoot mottle virus were found in Ohio wheat samples [9]. BMV was recently verified to be in Kansas wheat samples co-infecting with other viruses in our study [11].

Frequent monitoring and characterization of viral field populations provides valuable information about new isolates, dominant or emerging combinations, or novel viruses. Deployment of mite- and virus-resistant varieties is common in central and western Kansas, which can impose selection pressure on virus field populations. Three wheat streak mosaic resistance genes, *Wsm1*, *Wsm2*, *and Wsm3* have been identified [12,13,14,15]. *Wsm1* and *Wsm2* have been incorporated into several cultivars, including “Mace” [16], “Joe”, “Guardian”, and “Snowmass” [17,18]. However, both *Wsm1* and *Wsm2* exhibit temperature sensitivity, with resistance decreasing at temperatures above 20 °C [19,20]. *Wsm3* confers resistance to WSMV and TriMV as high as 24 °C [21,22]. Kansas farmers often blend these cultivars to thrive under viral pressure. Traditionally, infections are first diagnosed by visual symptoms; samples are then taken and confirmed using techniques such as serological tests and reverse-transcription polymerase chain reaction (RT-PCR) based on known virus antibodies and nucleotide primers and probes. Consequently, these methods are confounded to previously known viruses and are often not multiplexed, leading to multiple tests per sample. Throughout the Great Plains region, it is common for producers or local agriculture professionals to collect wheat tissue displaying infection and send it to their state diagnostic lab. for identification. Thereafter, diagnosticians use the techniques described above and return a report to the grower that may be used for educational or insurance purposes. Testing for each virus individually is time-consuming. Oxford Nanopore sequencing technology (ONT) is a commonly used cutting-edge technology due to its portability, rapid results, accurate identification of multiple pathogens, and short library preparation time [23]. This technology has been used as a surveillance tool for detecting fungal, bacterial [24], as well as plant viral pathogens [25]. While ONT is currently expensive and requires a high level of technique to perform, there is hope that this could become an adaptable platform to meet these diagnostic needs for simultaneous testing.

Surveys of wheat viruses have been conducted in many states of Great Plains [9,26] and exclusively in Kansas [10,27]. *Luteovirus* spp. and WSMV were the most prevalent co-occurring viruses across the state [10]. Additionally, combinations of two or more viral co-infections, including WSMV, TriMV, HPWMoV, BYDV, SBWMV, CYDV-RPV, and WSSM, have been reported [10,26]. However, these surveys were based on ELISA sample detection of only known viruses. A study carried out by [27] using a targeted RT-PCR and high-throughput Illumina short-read sequencing reported that WSMV single infection was prominent, followed by the mixed infection of WSMV and TriMV. They found only 1 sample with three viruses (WSMV + TriMV + HPWMoV) out of 98 infected samples.

High-throughput long-read sequencing offers a powerful, cost-effective, and precise method for characterizing large numbers of field samples, conducting metagenomics studies, and advancing future plant disease diagnostics. Comparing whole-genome (complete or near-complete) sequences among isolates assembled from long-read sequences enhances the likelihood of describing genomic variability without short-read/PCR bias. Short reads are prone to assembling chimeras of different viruses, which is very common in virus variants during de novo assembly [28,29]. ONT generates long reads that mitigate biases that commonly arise in metagenomics studies [29]. In this study, we reported a diverse wheat virus population and assembled long-read sequences using ONT for phylogenetic and recombination analysis

## 2. Materials and Methods

### 2.1. Winter Wheat Field Survey

A survey was conducted in major wheat-growing counties of Kansas during the wheat-growing seasons from May to July 2019, 2020, and 2021. Plants displaying virus-like symptoms, mimicking those that would be received in the diagnostic lab, and characterized by yellow discoloration and/or streaking or mosaic patterns on leaves, were sampled during the stem elongation and head development growth stages (Feekes 6 to 10). A total of 84 symptomatic wheat leaves (46 in 2019, 25 in 2020, and 13 in 2021) were collected from winter wheat fields in 47 different counties of Kansas. Leaf tissue of each sample was stored at −20 °C until the tissue could be processed for RNA extraction.

### 2.2. RNA Extraction and Nanopore Sequencing

Total RNA from each sample (200 mg of tissue) was extracted using a *mir*Vana miRNA extraction kit (Ambion Catalog number: AM1560, Thermo Fisher Scientific, Waltham, Waltham, MA, USA) according to the manufacturer’s instructions. A 7 µg quantity of total RNA was treated with 1 µL of DNase (Turbo DNase-Free ^TM^ kit; AM 1907, Ambion^®^, Thermo Fisher Scientific, MA, USA) according to the manufacturer’s instructions in a 50 µL reaction incubating at 37 °C for 30 min. RNA concentration was measured by a NanoDrop spectrophotometer (NanoDrop Technologies, Rockland, DE, USA). Reverse-transcription, strand-switching, PCR, barcoding, as well as bioinformatics analysis were described previously [11].

### 2.3. Sequence Alignment and Diagnosis

A collection of wheat virus genomes, focusing primarily on those already known and commonly tested for in the region, was gathered to use as a reference set for comparison (Supplementary Table S1). Barcoded trimmed sequence was aligned to the reference virus set using CLC Genomic Workbench^®^ v21.0.4 (Qiagen, MD, USA) with the following parameters: masking mode = no masking, match score = 1, mismatch cost = 2, cost of insertions and deletions = linear gap cost, insertion cost = 3, deletion cost = 3, length fraction = 0.5, similarity fraction = 0.8, global alignment = no, non-specific match handling = map randomly, output mode = create stand-alone read mappings, create report = yes, collect unmapped reads = no. Whole genome assemblies were based on consensus from the alignments. Incomplete genome assemblies were compared to the NCBI nucleotide (blastn) database to identify virus similarities. A minimum assembly length of 1000 bp was used.

### 2.4. Phylogenetic Analysis

Phylogenetic relationships of WSMV isolates were determined with the muscle program within Mega X [30]. Putative recombinants were excluded to reduce the conflicting phylogenetic signals [31]. Oat necrotic mottle virus (for WSMV), sugarcane streak mosaic virus and Caladenia virus A (for TriMV), and raspberry leaf blotch virus (for HPWMoV) were used as outgroups to root the trees. The best-fit nucleotide substitution models were determined by the maximum likelihood [30,32] necessary for constructing the phylogenetic tree and selected based on the lowest Akaike information criterion (AIC) and Bayesian information criterion (BIC) scores [33]. Maximum likelihood phylogenetic trees were constructed using Mega X with parameters as follows: number of bootstrap replications of 1000, nucleotide substitution model as mentioned above for different viruses, number of threads of 4.

### 2.5. Recombinant Analysis

Whole-genome consensus sequences of WSMV (Supplementary Table S2) and the complete reference genome sequences obtained from GenBank (Supplementary Table S3) were aligned using muscle alignment in Mega X [30]. Seven different algorithms were used in the RDP5 program [34] to examine the recombinant isolates. These algorithms were RDP [35], Bootscan [36], GENECONV [37], MaxChi [38], 3SEQ [39], Chimaera [40], and SiScan [41]. Putative recombinants and potential parents were determined if at least four out of seven algorithm methods were significant (*p* < 0.01).

## 3. Results

An average of 4.48 × 10^5^ raw reads was obtained after mapping to a cereal virus reference genome database. An average of 9.78 × 10^3^ reads of WSMV were obtained after mapping with a reference sequence with an average coverage of 991.82 X (Supplementary Table S2). For TriMV, an average of 6.06 × 10^3^ reads with an average coverage of 301.56 X was obtained after mapping with the reference genome (Supplementary Table S4). Complete and near-complete genomes, one RNA1 and RNA2, four RNA3A, three RNA3B, four RNA4, one RNA5, two RNA6, four RNA7, and two RNA8, of HPWMoV with an average of 4.5 × 10^3^ long reads with an average coverage of 1126.31 X were obtained and deposited in the GenBank (Supplementary Table S5). Two complete genomes of RNA1 and RNA2 with an average of 6.93 × 10^3^ long reads with an average coverage of 410.71 X (Supplementary Table S6) of SWMV were obtained. Coverage of BYDV and WSSMV was insufficient to produce useful full genome sequences and were omitted from GenBank submission.

**Figure 1:**
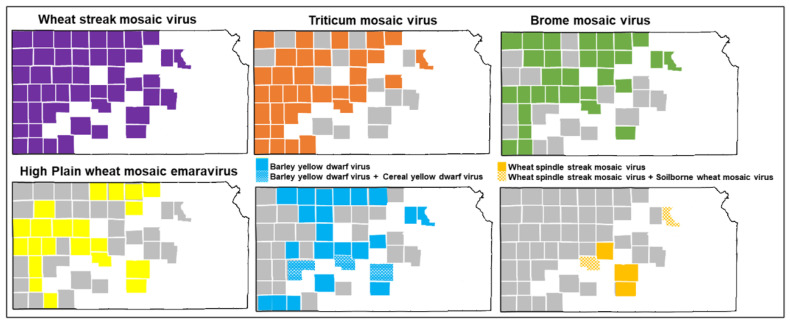
Map of Kansas counties with the viruses identified in this study using Oxford Nanopore sequencing. Virus-like symptomatic wheat leaves were collected from the field in 2019, 2020, and 2021. White area of the map indicates never sampled, gray area indicates sampled but negative result of a virus and colored area indicates positive result of a virus.

At least one virus was detected in all 84 total samples as we sampled the wheat leaves that showed the virus-like symptoms of mosaic, chlorosis, and stunting. A total of eight different wheat viruses, *T. tritici* (WSMV), *P. tritici* (TriMV), *B. BMV* (BMV), *E. tritici* (HPWMoV), *L. pavhordei* (BYDV), *L. sgvhordei* (CYDV), *B. tritici* (WSSMV), and *F. tritici* (SBWMV), were detected (Table 1). Figure 1 illustrates the distribution of wheat viruses across the major wheat-growing counties of Kansas. WSMV was the most dominant virus identified in all 47 counties sampled, followed by TriMV in 33 counties and BMV in 30 counties (Figure 1). BYDV and HPWMoV were identified in 22 and 21 counties, respectively. WSSMV, CYDV, and SBWMV were found in five, three, and two counties, respectively (Figure 1).

**Figure 2:**
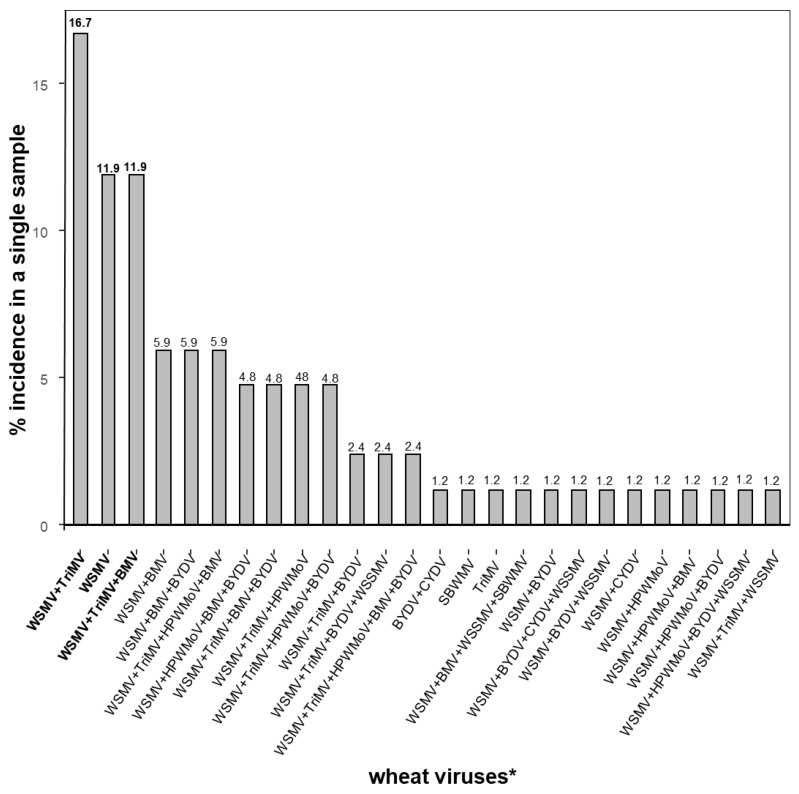
Percent incidence of wheat viruses *: wheat streak mosaic virus (WSMV), Triticum mosaic virus (TriMV), High Plains wheat mosaic emaravirus (HPWMoV), brome mosaic virus (BMV), barley yellow dwarf virus (BYDV), wheat spindle streak mosaic virus (WSSMV), cereal yellow dwarf virus (CYDV), soilborne wheat mosaic virus (SBWMV), and virus combinations in samples collected from Kansas wheat fields detected through Oxford Nanopore sequencing. Virus-like symptomatic wheat leaves were collected from the field in 2019, 2020, and 2021.

The survey showed that most of the wheat samples were co-infected with two to five viruses, while single virus infections were the exception. The most dominant co-infection was WSMV + TriMV (16.7%), followed by WSMV + TriMV + BMV (11.9%) and WSMV only (11.9%) (Figure 2). One sample was infected with TriMV only, but TriMV was found co-infected with all other viruses except SBWMV. BMV was found to be co-infected with all other wheat viruses, however most commonly co-infected with WSMV, TriMV, and HPWMoV (27.8%) [11]. Five viruses (WSMV + TriMV + HPWMoV + BMV + BYDV) were found in a single sample. WSSMV was co-infected in various combinations of the other viruses. There was a single sample where SBWMV was co-infected with WSSMV, WSMV, and BMV (Figure 2). SBWMV was found only in Pawnee and Riley counties; however, WSSMV was found in Barton, Kingman, and Reno counties along with Pawnee and Riley counties (Figure 2).

### 3.1. Sequence Alignments

**WSMV:** Virus titer was high enough in most of the WSMV samples to produce full-length consensus assemblies, which were named based on the county and year of origin. These assemblies were compared to other WSMV genomes. Isolates showed nucleotide identity ranges from 97.0% to 98.2% with WSMV type (AF285169.1) except for three isolates 19RH1 having only 88.4%, 19SV 90.4%, and 20GO 94.5% nucleotide identity with the WSMV-type strain (Supplementary Table S2). Most isolates showed lower nucleotide identity (~88%) with Central European isolates. However, 19RH1 showed >97%, 19SV showed >95%, and 20GO showed ≥92% nucleotide identity with central European isolates. 19RH1 had an in-frame three-nucleotide GCA deletion at nucleotide position 8412 to 8414 of coat protein, leading to the loss of a glycine residue at position 2761 in the polyprotein.

**TriMV:** The whole-genome sequences of 11 isolates TriMV were assembled with the complete genome sequence of the TriMV retrieved from the GenBank. TriMV isolates from this study showed high nucleotide identity of 99.6 to 99.7% with TriMV KS isolate (FJ263671.1) (Supplementary Table S4). The changes occurred randomly and vary among isolates across the genome.

**HPWMoV:** The complete and near-complete sequences of eight RNA segments of HPWMoV isolates obtained in this study were aligned with reference genomes and other published isolates retrieved from GenBank (Supplementary Table S7). All RNA segments had high nucleotide identity (>99%) with reference sequences, but 20MC2 RNA3A and RNA7 sequences showed 96.3% and 96.6% nucleotide identity, respectively (Supplementary Table S5). Isolate 20RH2 RNA7 showed only 85.5% nucleotide identity with the RNA7 reference sequences (Supplementary Table S6). Two variant sequences of RNA 3A and RNA 3B were found. RNA 3A encodes 286 amino acids of 33.2 kDa, and RNA 3B encodes for the 289 amino acids of 33.4 kDa nucleocapsid proteins. RNA 3A and RNA 3B variants found in this study had an average of within group and between group percent identity 12.43% sequence divergence between these variants. The alignment of protein sequences of 3A and 3B obtained from this study and with sequences retrieved from GenBank showed a 3-amino-acid insertion in RNA 3B at the positions of 23, 24, and 287 C-terminus of the protein in all isolates used (Supplementary Figure S1). These insertions differentiate RNA 3B from 3A.

**SBWMV:** The complete sequences of both RNA1 and RNA2 of SBWMV isolates obtained from Pawnee and Riley counties were aligned with RNA1 and RNA2 reference sequences separately (Supplementary Table S7). RNA1 sequences of SBWMV samples from Pawnee and Riley County had more than 98% and 96% nucleotide identity compared with the reference sequences, respectively. RNA2 sequences of both isolates had more than 98% nucleotide identity with the reference sequences (Supplementary Table S6). RNA1 of Riley and Pawnee County isolates encodes three proteins: measuring 149.9/150 kDa (from 102 to 4064 nt, 1320 amino acids), 54.7/54.6 kDa (from 4185 to 5588, 467 amino acids), and 37.2/37.3 kDa (5653–6636, 327aa), respectively. RNA1 of Riley and Pawnee County isolates consists of 7096 and 6995 nucleotides, respectively. The reference genome was 7099 nucleotides long; around 100 bps were missing from the 3′ untranslated region of Pawnee County isolates. RNA2 is the shorter particle. Both counties’ isolates contain 3590/3591 nucleotides and encode for three proteins: measuring 19.3 kDa (from 334 to 864 nt, 176 amino acids), 54.0 kDa (from 1141 to 2598, 458 amino acids), and 18.8 kDa (from 2665 to 3189, 174 amino acids).

### 3.2. Recombinant Analysis of WSMV

Of the total samples, 37.8% of WSMVs (14 out of 37) were identified as potential recombinants by at least five algorithms of the RDP5 program at a significant value of *p* < 0.05 (Supplementary Table S8). These algorithms also provided the potential major and minor parents. These recombinant isolates were 19SV, 19ST, 19RA3, 19TR1, 19FI, 19GH1, 10SW, 20GO, 20WA, 20EW, 20TR2, 20GH2, and 20JW3.

### 3.3. Phylogenetic Analysis

**Figure 3:**
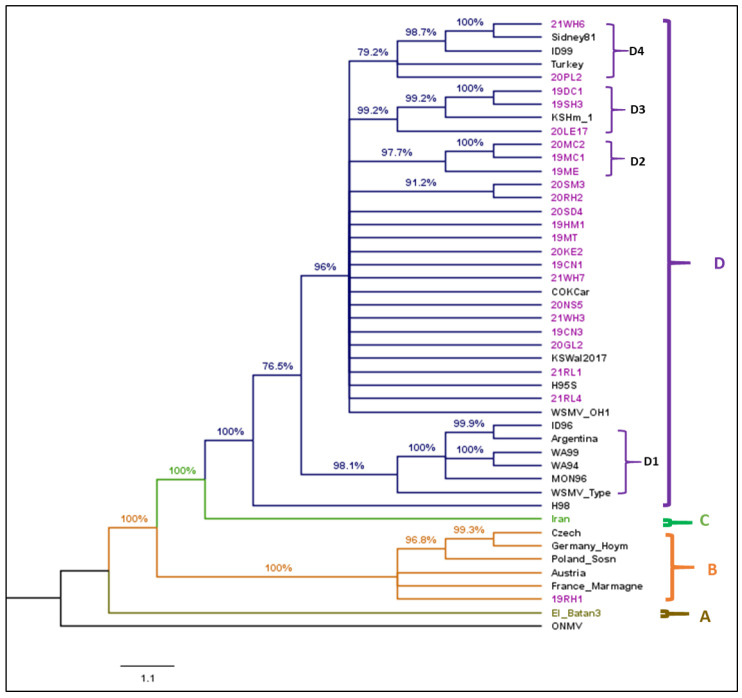
Cladogram of wheat streak mosaic virus (WSMV) isolates sequenced in this study (heightened in purple text) and selected strains. The phylogenetic tree was made with maximum likelihood analysis with a GTR + G + I substitution model of nucleoprotein sequence with 1000 bootstrap. The tree with the highest log likelihood (−56,327.64) is shown. The percentage of trees in which the associated taxa were clustered is shown next to the branches. The posterior probability of 70% was the cutoff value and branches not supported were collapsed. Oat necrotic mottle virus was used as an outgroup in the analysis. Brackets on the right side indicate the taxa clustered in WSMV clades A to D. Clade D is further divided into subclades D1 to D4. Purple color represents samples from this study, other colors represent different clades.

**WSMV:** The complete nucleoprotein or coding sequence of 23 WSMV isolates (9 from 2019 and 2020, 5 from 2021) from this survey and 22 reference isolates obtained from GenBank (Supplementary Table S4) were used to construct a cladogram. Constructing a cladogram without recombination analysis leads to conflicting phylogenetic signals [31]. Therefore, before phylogenetic analysis, recombinant analysis was performed, and 14 potential recombinant isolates detected by the RDP5 program were excluded from phylogenetic analysis. Oat necrotic mottle virus (ONMV) was used as an outgroup. The cladogram constructed from 45 complete nucleoprotein sequences of WSMV isolates consists of four main clades (Figure 3). The best-fit nucleotide substitution model determined by maximum likelihood for WSMV sequences was GTR + G + I (General Time-Reversible model with Gamma-distributed and Invariant sites).

Clade A represents isolate ‘El-Batán’ from Mexico. Clade B includes European isolates characterized by a deletion of the glycine residue at position 2761 in the coat protein region because of the deletion of the GCA codon at nucleotide positions 8412 to 8414. The isolate (19RH1) collected from Rush County of KS was clustered with European isolates. Clade C represents an isolate from Iran. Clade D includes isolates from the United States, Argentina, and Turkey. Clade D isolates were further divided into four subclades (D1 to D4). D1 contained isolates from the American Pacific Northwest and WSMV type isolate, D2 was constituted by only isolates from this study. Isolates from Colorado, other already detected Kansas isolates and other Kansas isolates from this study comprised a small group and polytomies between sub-clades D1 and D2. D3 also contained the isolates from Kansas only with one already detected Kansas isolate. D4 included isolates from Kansas, Nebraska, Idaho, and Turkey.

**Figure 4:**
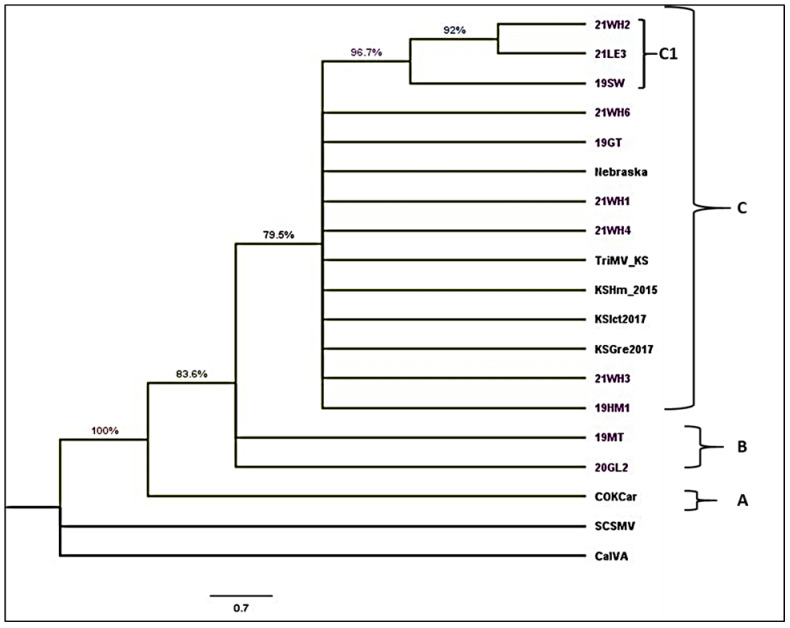
Cladogram of Triticum mosaic virus (TriMV) isolates sequenced in this study (heightened in purple text) and selected strains. TriMV isolates are divided into four clades A to C and clade C with C1 sub-clade. The phylogenetic tree was made with maximum likelihood analysis with a GTR + G substitution model of nucleoprotein sequence with 1000 bootstrap. The tree with the highest log likelihood (−25,213.21) is shown. The percentage of trees in which the associated taxa were clustered is shown next to the branches. The posterior probability of 70% was the cutoff value and branches not supported were collapsed. Sugarcane streak mosaic virus and Caladenia virus A were used as outgroups in the analysis. Purple color represents samples from this study, other colors represent different clades. **HPWMoV:** The coding sequence of HPWMoV nucleocapsid protein RNA3 and its two variants, RNA3A and RNA 3B were used to construct a cladogram (Figure 5). Five RNA3, three RNA3A, and RNA3B nucleocapsid protein sequences obtained from GenBank (Supplementary Table S5), four RNA3A, and three RNA3B sequences obtained from this study were included in the cladogram. Raspberry leaf blotch virus (RLBV) RNA3 nucleoprotein sequence was used as an outgroup. The best-fit nucleotide substitution model determined by maximum likelihood for HPWMoV sequences was T92 + G (Tamura-3-parameter with Gamma distributed rate). Because of the 95–99% within-group sequence identity and 87–89% between-group identity, RNA3 clustered separately in the middle of the cladogram between RNA3A and RNA3B. RNA3A isolates from this study and previously sequenced Nebraska and Kansas isolates were clustered together with a common node of significant bootstrap support (Figure 5). RNA3B GG1 Ohio isolates form a separate cluster. However, RNA3B isolates from this study (20MC2 and 20SC2) and previously sequenced Kansas isolate (KS7) clustered together. One Nebraska isolate, and 20KE2 isolate, formed a single polytomy within the RNA3B cluster.

**TriMV:** The cladogram was constructed using a complete nucleoprotein sequence with 11 TriMV Kansas isolates from this study (Supplementary Table S3) and 6 TriMV isolates from sequences retrieved from GenBank (Supplementary Table S4). Sugarcane mosaic virus (YN-YZ211) and Caladenia virus A (CalVA KP1) were used as outgroups. The best-fit nucleotide substitution model determined by maximum likelihood for TriMV sequences was GTR + G (General Time Reversible model with Gamma distributed rate).The topology of the cladogram consists of three clades: A, B, and C (Figure 4). Clade A consisted of single isolates from Colorado. Clade B contained two isolates, 19 MT and 20GL2, collected during this study. Clade C consisted of one isolate from Nebraska and four isolates previously collected from Kansas and 9 isolates collected during this study. Clade C comprises one subclade C1, including two isolates from Wichita and Lane County collected in 2021 and one from Seward County, Kansas collected in 2019.

## 4. Discussion

Plant viral diagnostics become intricate due to mixed infections of multiple viruses. Accurate diagnosis of plant viruses is essential for reducing disease spread and effective management. This study identified positive-sense ssRNA viruses, bipartite positive-sense RNA viruses, a tripartite positive-sense RNA virus, and an octapartite negative-sense RNA virus in a single sample. Co-infection of plant viruses within a single plant is common, resulting in a synergistic negative impact on the host [45,46,47]. For instance, maize lethal necrosis, maize chlorotic mottle virus, and sugarcane mosaic virus cause synergistic impacts [48]. Previous research has demonstrated synergistic interactions between isolates of casava mosaic virus [49]. Co-infection has also been found to increase vector transmission efficiency and systemic movement, providing a fitness advantage due to the synergistic effect of co-infection and high titers of WSMV and TriMV [50,51]. Additionally, the transmission efficiency of TriMV and HPWMoV mixed infection with WSMV was higher compared to a single infection of these viruses [50,52]. Mixed infection results in mutual benefits between co-infected viruses and leads to a more significant impact on yield loss. Greenhouse and field studies have shown significant yield loss due to the co-infection of WSMV and TriMV [53,54]. In this study, we targeted wheat RNA viruses by using Oligo (dT) primers and could identify viruses with “multiple adenylation” in the genome other than viral genomes with poly (A) tails and limited the scope of identification of other potential wheat DNA viruses.

Oxford Nanopore sequencing (ONT) presents significant potential as a tool for identifying viral diseases in wheat field samples [25] positioning itself as a potential diagnostic method. Aside from disease surveillance, ONT is a powerful choice for viral metagenomics studies. ONT generates long reads that mitigate biases that commonly arise in metagenomics studies [29]. Short-read sequencing platforms often introduce biases, such as the generation of chimeras from short reads of different virus variants during de novo assembly [29,30]. In contrast, the long reads of ONT decrease the likelihood of unassigned reads during contig assembly and enhance the probability of obtaining true, unbiased genetic variability. This is achieved by adjusting requirements during sequencing and post-sequencing of the bioinformatics pipeline [55]. The availability of high-accuracy base calling packages, new flow cells, and downstream computational methods are available to correct ONT sequencing data for deep sequence analysis and metagenomics [56]. In this study, we also compared the whole-genome consensus sequence of WSMV constructed after getting short-read sequences from Illumina short-read and long-reads from nanopore sequencing; we found no difference in genome. The average reads of virus samples and coverage of each sample were varied, possibly due to sensitivity of the RNA degradation during sample collections from the fields and storage of the samples or due to the different levels of virus accumulation.

Understanding the viral population structure is crucial for the development or recommendation of more effective genetic resistance sources. We identified eight different viruses and co-infection of one or more viruses is common and co-infection of WSMV and TriMV was most dominant, followed by WSMV + TriMV + BMV. This knowledge is useful for developing breeding programs because the *Wsm2* resistance gene confers resistance to WSMV alone but not to the other viruses. Future breeding programs should pivot toward screening cultivars for multiple viruses that represent the actual field condition. The process of selecting virus-resistant cultivars through varietal screening programs needs to be dynamic, guided by frequent monitoring and characterization of field viral populations. Screening nurseries can adopt strategies such as mechanical inoculation with multiple viruses or establishing breeding nurseries in fields with a history of consistent natural infections. To optimize natural inoculation with field viral populations, one can establish the nursery next to a field with volunteer wheat or simulate the volunteer wheat around the varietal screening nursery.

Some samples of this study were collected from cultivars with known resistance genes from Lane and Wichita counties. Notably, wheat cultivars with *Wsm2* resistance gene such as “Joe” and “Guardian” carrying *Wsm2* + curl mite resistance gene exhibited heavy infections with a high virus load. The counties where WSM complex viruses and Brome mosaic virus were identified coincided with the regions where these varieties are adapted. Both “Joe” and “Guardian” were infected with WSMV and TriMV. This finding prompts further investigation into whether the susceptibility of these varieties to infection is due to high temperatures, given the temperature sensitivity of *Wsm2* resistance gene, where resistance is less effective at higher temperatures [19,20], or if resistant-breaking isolates of WSMV are present. One such *Wsm2* resistant-breaking isolate (KSH294) was already identified from foxtail in Hays, Kansas in 2019 [57], suggesting the possibility of similar isolates in the field.

The widespread deployment of wheat cultivars carrying a single resistance gene across a large geographic area intensifies the selection pressure on viruses. Phylogenetic analysis of WSMV isolates from Kansas revealed diversity, with isolates forming distinct clades and subclades. This phylogenetic relationship aligns with previous studies based on the coat protein sequence [58,59] and recent full-genome sequences [27]. The WSMV isolate collected from Rush County (19RH1) clustered within Clade B, alongside European isolates. European isolates were reported from the Pacific Northwest region of the United States [60] and from the Great Plains [27]. This diversity, encompassing European isolates and putative recombinants in Kansas, holds implications for breeding programs, as different WSMV isolates may interact differently with resistance genes. The presence of genetically variable WSMV isolates in Kansas increases the potential for the evolution of resistance-breaking isolates, emphasizing the need for breeding strategies that incorporate tolerant cultivars or those with a stack of minor and major resistance genes to alleviate selection pressure in viral population breeders should consider using tolerant cultivars or cultivars with a stack of minors and major resistance genes to reduce the selection pressure in viral populations.

In this study, recombinant analysis revealed that about 38% of the characterized isolates exhibited recombination, with putative recombinant isolates positioned with European (Clade B) and United States (Clade D) isolates as the major and minor parents (Supplementary Table S8). This result raises concerns, considering the *Wsm2* resistant-breaking isolate (KSH294) was reported as a potential recombinant in a previous study [57]. The presence of recombinant isolates in the field indicates the significant role recombination plays in the evolution of WSMV strains, potentially impacting infections in wheat cultivars with resistance genes. Additionally, the possibility of an expanded host range or increased aggressiveness or virulence cannot be disregarded. However, there was no information of host resistance genes for those recombinant isolates reported in this study. Recombination analysis provides insights into whether it serves as a major evolutionary force in the field population of WSMV. This analysis aids in identifying the putative recombinant isolates, allowing for phylogenetic analysis to be conducted without them. This approach minimizes conflicting phylogenetic signals during analysis. Recombination, a prevalent phenomenon in plant viruses providing evolutionary advantage, has been observed in various potyviruses. For example, multiple recombinants were reported from plum pox virus [61], isolates of potato potyvirus Y [62,63], bean common mosaic virus, zucchini yellow mosaic virus [63], and watermelon mosaic virus [64]. Understanding the virus–host interaction requires future studies to systematically survey wheat cultivars with known resistance genes or defense mechanisms.

This study successfully obtained the whole-genome sequence of all eight segments of HPWMoV from different Kansas counties, revealing notable distinctions in RNA3 variants. Specifically, RNA3B exhibited greater diversity compared to RNA3A, aligning with previous findings in Ohio isolates [65] and Nebraska isolates [66]. The sequence divergence observed within and between RNA3A and 3B, along with phylogenetic relationship, demonstrated a higher similarity between Kansas and Nebraska isolates than Ohio isolates. However, RNA3A isolates from this study, Nebraska and Ohio, clustered together, indicating lower variability in RNA3A compared to RNA3B. The contrast in location-wise variability between HPWMoV RNA3A and RNA3B, as shown in this study, diverges from Stewart’s 2016 findings, suggesting an intriguing avenue for future comparisons. A comprehensive examination of HPWMoV isolates from different wheat cultivars, locations, and vector types would provide valuable insights into the overall variability of HPWMoV isolates.

The characterization of the whole genomes of RNA1 and RNA2 of SBWMV isolates in this study represent the first isolates from Kansas and serves as a vital molecular resource for accurate diagnosis and facilitates future phylogenetic and evolutionary studies of SBWMV. Accurate diagnosis of SBWMV is crucial due to the persistent nature of its vector, *Polymyxa graminis*, whose resting spores can remain dormant and invasive in soil for up to 30 years and can infect the host in favorable conditions [67]. Controlling this disease becomes challenging once it infects a wheat field. Therefore, the molecular characterization of SBWMV isolates not only ensures accurate diagnosis but also helps in the development of resistant cultivars through targeted breeding programs.

Overall, this study demonstrates the cost-effective potential of utilizing third-generation long reads from Oxford Nanopore sequencing technology for simultaneous analysis of multiple samples through barcoding. The comprehensive results unveiled wheat viruses across six families and eight genera, encompassing mono-, bi-, tri-, and octapartite positive to negative-sense RNA viruses in a single sample. The identified families and genera include *Potyviridae* (Genera *Tritimovirus*, *Poacevirus*, and *Bymovirus*), family *Fimoviridae* (Genus *Emaravirus*), family *Tombusviridae* (Genus *Luteovirus*), family *Virgaviridae* (Genus *Furovirus*), and family *Bromoviridae* (Genus *Bromovirus*). The significance of information on diverse wheat virus populations derived from this study also relays crucial implications for the development of cost-effective diagnostic tools for diagnostic laboratories, showing that a single sample analysis can yield the same results as five or more individual traditional tests.

## Figures and Tables

**Figure 5:**
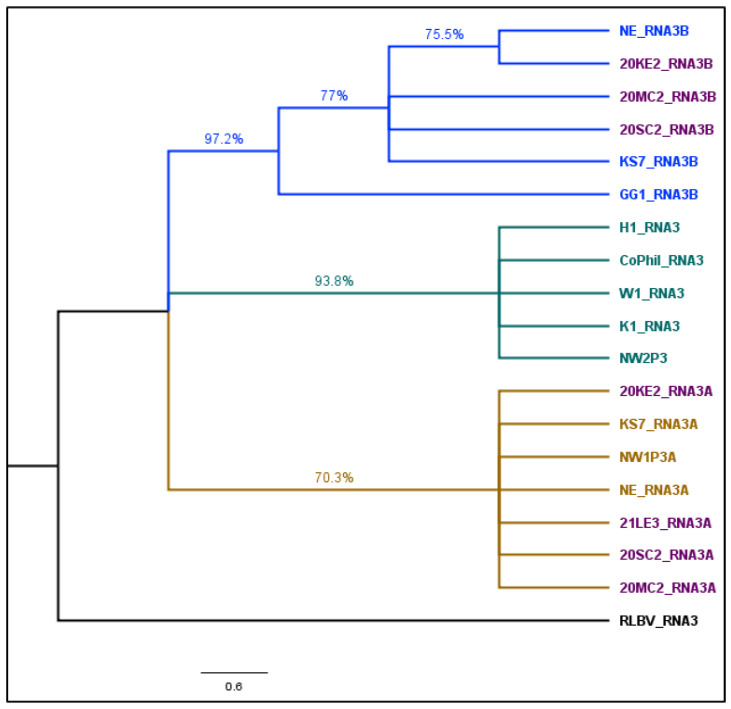
Cladogram of RNA3 of High Plains wheat mosaic emaravirus (HPWMoV) isolates sequenced in this study (heightened in purple text) and selected strains. The phylogenetic tree was made with maximum likelihood analysis with a T92 + G substitution model of nucleoprotein sequence with 1000 bootstrap. The tree with the highest log likelihood (−3324.14.21) is shown. The percentage of trees in which the associated taxa were clustered is shown next to the branches. The posterior probability of 70% was the cutoff value and branches not supported were collapsed. Raspberry leaf blotch virus (RLBV) was used as an outgroup in the analysis. Purple color represents samples from this study, other colors represent different clades.

**Table 1:** Taxonomy of the eight viruses identified using Oxford Nanopore sequencing. Taxonomic classification based on the International Committee on Taxonomy of Viruses (ICTV), Virus Taxonomy: 2023 Release, MSL #39 [42,43,44].

	Common Name							
**Category**	Wheat streak mosaic virus	Triticum mosaic virus	Brome mosaic virus	High Plain wheat mosaic emaravirus	Barley yellow dwarf virus	Cereal yellow dwarf virus	Wheat spindle streak mosaic virus	Soilborne wheat mosaic virus
**Realm**	Riboviria	Riboviria	Riboviria	Riboviria	Riboviria	Riboviria	Riboviria	Riboviria
**Kingdom**	Orthornavirae	Orthornavirae	Orthornavirae	Orthornavirae	Orthornavirae	Orthornavirae	Orthornavirae	Orthornavirae
**Phylum**	Pisuviricota	Pisuviricota	Kitrinoviricota	Negarnaviricota	Kitrinoviricota	Kitrinoviricota	Pisuviricota	Kitrinoviricota
**Class**	Stelpaviricetes	Stelpaviricetes	Alsuviricetes	Bunyaviricetes	Tolucaviricetes	Tolucaviricetes	Stelpaviricetes	Alsuviricetes
**Order**	Patatavirales	Patatavirales	Martellivirales	Elliovirales	Tolivirales	Tolivirales	Patatavirales	Martellivirales
**Family**	Potyviridae	Potyviridae	Bromoviridae	Fimoviridae	Tombusviridae	Tombusviridae	Potyviridae	Virgaviridae
**Genus**	*Tritimovirus*	*Poacevirus*	*Bromovirus*	*Emaravirus*	Luteovirus	*Luteovirus*	*Bymovirus*	*Furovirus*
**Species**	*Tritimovirus tritici*	*Poacevirus tritici*	*Bromovirus BMV*	*Emaravirus tritici*	*Luteovirus pavhordei*	*Luteovirus sgvhordei*	*Bymovirus tritici*	*Furovirus tritici*

## Data Availability

The datasets presented in this study can be found in online repositories. The names of the repository and accession numbers can be found below: https://www.ncbi.nlm.nih.gov/ (accessed on 1 January 2025)., SRA PRJNA915982.

## Supplementary Materials

The following supporting information can be downloaded at: https://www.mdpi.com/article/10.3390/v17010126/s1, Figure S1: Alignment of nucleocapsid protein sequence encoded by High Plains wheat mosaic emaravirus RNA3 and its two variant RNA3A and 3B isolates obtained from this study as well as isolates sequence retrieved from GenBank. RNA3B showed a 3 amino-acid insertion at the positions of 23, 24, and the 287 C terminus of the protein; Table S1: List of sequences of cereal viruses retrieved from GenBank that were used as reference genomes to get consensus sequences; Table S2: List of complete viral genome sequences and characterization of the consensus sequences of wheat streak mosaic virus that identified on wheat samples using Nanopore sequencing; Table S3: List of sequences of viruses retrieved from GenBank; Table S4: List of complete viral genome sequences and characterization of the consensus sequence of Triticum mosaic virus identified on wheat samples using Nanopore sequencing; Table S5: List of complete viral genome sequences and characterization of the consensus sequences of High Plains wheat mosaic emaravirus identified on wheat samples using Nanopore sequencing; Table S6: List of complete viral genome sequences and characterization of the consensus sequence of Soilborne wheat mosaic virus identified on wheat samples using Nanopore sequencing; Table S7: List of sequences of High Plains wheat mosaic emaravirus (HPWMoV) isolates retired from GenBank; Table S8: Potential recombinant isolates of wheat streak mosaic virus (WSMV) analyzed by using 7 different algorithms in the RDP5 program.
