# Supplementary material for: High-Throughput Oxford Nanopore Sequencing Unveils Complex Viral Population in Kansas Wheat: Implications for Sustainable Virus Management
Source: Viruses. 2025 Jan 17;17(1):126. doi: 10.3390/v17010126 (PMC11768895; doi:10.3390/v17010126)
Supplement: Supplementary file 1 [file viruses-17-00126-s001.zip › viruses-3412810-supplementary.pdf]

Supplementary Table 1. List of sequences of cereal viruses retrieved from GenBank that were used as reference genomes to get consensus sequences.

Name of virus	Accession number
Ageratum yellow leaf curl betasatellite	KC305091.1
Agropyron mosaic virus	NC_005903.1
Barley mild mosaic virus	AJ544268.1
Barley stripe mosaic virus RNA1	NC_003469.1
Barley stripe mosaic virus RNA2	NC_003481.1
Barley stripe mosaic virus RNA3	NC_003478.1
Barley yellow dwarf virus-PAS	NC_002160.2
Barley yellow dwarf virus-MAV	NC_003680.1
Barley yellow dwarf virus-GAV	KF523382.1
Barley yellow dwarf virus-PAV	EF043235.1
Barley yellow striate mosaic virus, polymerase (L) gene	FJ665628
Barley yellow striate mosaic virus, glycoprotein (G) gene	KP163565.1
Cereal yellow dwarf virus	EF521830.1
Hordeum mosaic virus	NC_005904.1
Maize streak virus	AF239960.1
Oat golden stripe virus RNA1	NC_002358.1
Oat golden stripe virus RNA2	NC_002357.1
Oat necrotic mottle virus	NC_005136.1
Tobacco mosaic virus Queensland	AF332868
Rice black streaked dwarf virus (S1)	KC134289.1
Rice black streaked dwarf virus (S2)	KC134290.1
Rice black streaked dwarf virus (S3)	KC134291.1
Rice black streaked dwarf virus (S4)	KC134292.1
Rice black streaked dwarf virus (S5)	KC134293.1
Rice black streaked dwarf virus (S6)	KC134294.1
Rice black streaked dwarf virus (S7)	KC134295.1
Rice black streaked dwarf virus (S8)	KC134296.1
Rice black streaked dwarf virus (S9) cds_AFX68415.1_1	KC134297.1
Rice black streaked dwarf virus (S9) cds_AFX68415.1_2	KC134297.1
Rice black streaked dwarf virus (S10)	KC134298.1
Soil borne wheat mosaic virus RNA1	KT736088.1
Soil borne wheat mosaic virus RNA2	KT736089.1
Wheat streak mosaic virus type strain	AF285169
Wheat streak mosaic virus Hoym	HG810954.1
Wheat mosaic virus KS7 RNA1	KT988860.1
Wheat mosaic virus KS7 RNA2	KT988861.1
Wheat mosaic virus KS7 RNA3A	KT988862.1
Wheat mosaic virus KS7 RNA3B	KT988863.1
Wheat mosaic virus KS7 RNA4	KT988864.1
Wheat mosaic virus KS7 RNA5	KT988865.1
Wheat mosaic virus KS7 RNA6	KT988866.1
Wheat mosaic virus KS7 RNA7	KT988867.1
Wheat mosaic virus KS7 RNA8	KT988868.1

Name of virus	Accession number
Wheat dwarf virus	KJ473705.1
Wheat eqlid mosaic virus	NC_009805.1
Wheat rosette stunt virus	AF059602.1
Wheat spindle streak mosaic virus RNA1	NC_040508.1
Wheat spindle streak mosaic virus RNA2	NC_040507.1
Wheat stripe virus RNA2	AY312434.1
Wheat stripe virus RNA3	AY312435.1
Wheat stripe virus RNA4	AY312436.1
Wheat yellow mosaic virus RNA1	AB910332.1
Wheat yellow mosaic virus RNA2	AB910336.1
Foxtail mosaic virus	EF630359.1
Triticum mosaic virus KS	FJ263671.1
Barley virus G	KT962089.1
Barley yellow mosaic virus RNA1	AJ132268.1
Barley yellow mosaic virus RNA2	AJ132269.1
Brome mosaic virus RNA1	NC_002026.1
Brome mosaic virus RNA2	NC_002027.1
Brome mosaic virus RNA3	NC_002028.2
European wheat striate mosaic virus RNA1	MN044342.1
European wheat striate mosaic virus RNA2	MN044343.1
European wheat striate mosaic virus RNA3	MN044344.1
European wheat striate mosaic virus RNA4	MN044345.1
Chinese wheat mosaic virus RNA1	NC_002359.1
Chinese wheat mosaic virus RNA2	NC_002356.1
Johnsongrass mosaic virus	KX897165.1
Maize chlorotic mottle virus	X14736.2
Maize yellow dwarf virus-RMV	KC921392.1
Maize yellow mosaic virus-Morogoro	MW036244.1
Maize yellow striate virus	KY884303.1
Oat dwarf virus	KX533459.1
Panicum mosaic virus	MH885652.1
Ryegrass mosaic virus	MT005828.1
Sitobion miscanthi flavi-like virus	MH778148.1
Soil borne cereal mosaic virus RNA1	NC_002042.1
Soil borne cereal mosaic virus RNA2	NC_002041.1
Sugarcane mosaic virus	AJ297628.1
Wheat leaf yellowing-assocaited virus	KY605226.1
Wheat yellow dwarf virus-GPV	NC_012931.1
Wheat yellow striate virus	MG604920.1
Brome streak mosaic rymovirus	Z48506.1

Supplementary Table 2. List of complete viral genome sequences and characterization of the consensus sequences of wheat streak mosaic virus that identified on wheat samples using Nanopore sequencing.

Sample ID	County	Accession number	No of reads†	Coverage (X)	Nucleotide identity (%)‡
19CN1	Cheyenne	OR900886	8232	334.28	98.0
19DC1	Decatur	OR900888	32941	1141.6	97.2
19CN3	Cheyenne	OR900889	4504	234.8	97.8
19SH3	Sherman	OR900909	15240	2820.36	97.3
19SV	Stevens	OR900919	3929	141.3	90.4
19ST	Stanton	OR900918	3590	179.39	97.0
19NS2	Ness	OR900917	12633	826.23	97.5
19RA3	Rawlins	OR900922	23855	4180.06	97.8
19GH1	Graham	OR900912	939	113.27	97.9
19SW	Seward	OR900898	4494	241.62	97.6
19TR1	Trego	OR900905	8708	383.8	98.1
19MC1	Mitchell	OR900916	6995	207.72	97.3
19FI	Finney	OR900901	5133	263.39	98.1
19RH1	Rush	OR900914	4483	327.94	88.4
19HM1	Hamilton	OR900900	8637	576.3	97.9
19ME	Meade	OR900895	4268	199.45	97.8
19MT	Morton	OR900896	28509	1752.32	98.4
20NS5	Ness	OR900906	7971	405.12	97.8
20PL2	Phillips	OR900902	1787	126.21	98.2
20SD4	Sheridan	OR900910	31250	5432.71	97.6
20GL2	Greeley	OR900908	16781	1449.28	98.2
20GO	Gove	OR900920	8357	307.58	94.9
20TR2	Trego	OR900907	3833	182.9	97.6
20GH2	Graham	OR900911	32426	7525.89	98.1
20WA	Wallace	OR900920	7400	258.44	96.3
20MC2	Mitchell	OR900903	2608	163.53	97.3
20JW3	Jewell	OR900904	5501	333.36	98.1
20EW	Ellsworth	OR900897	1033	93.7	96.9
20SM3	Smith	OR900887	1454	86.21	97.6
20KE2	Kearny	OR900899	2459	158.48	98.2
20LE17	Lane	OR900894	10029	681.0	98.2
20RH2	Rush	OR900891	4030	227.2	98.0
21WH6	Wichita	OR900890	11896	834.85	97.1
21WH7	Wichita	OR900892	7012	167.87	97.6
21WH3	Wichita	OR900915	3345	162.04	98.2
21RL4	Riley	OR900893	6224	430.08	97.4
21RL1	Riley	OR900913	17683	3746.95	97.3

† Number of reads obtained using nanopore sequencing and mapped with reference genome using CLC Genomics Workbench

‡Percent nucleotide identity of Kansas isolates sequence of this study to the type species (WSMV type isolate. AF285169.1) sequence

Supplementary Table 3. List of sequences of viruses retrieved from GenBank.

Sample ID	virus	Origin	Accession Number
Argentina	Wheat streak mosaic virus	Argentina	FJ348359.1
Austria	Wheat streak mosaic virus	Austria	LN624217.1
COKCar	Wheat streak mosaic virus	CO, USA	MT762110.1
Czech	Wheat streak mosaic virus	Czech	AF454454.1
El Batan3	Wheat streak mosaic virus	Mexico	AF285170.1
H95S	Wheat streak mosaic virus	KS, USA	AF5116114.2
H98	Wheat streak mosaic virus	KS, USA	AF511615.2
Germany_Hoym	Wheat streak mosaic virus	Germany	HG810954.1
ID96	Wheat streak mosaic virus	ID, USA	AF511618.2
ID99	Wheat streak mosaic virus	ID, USA	AF511619.2
KSHm1	Wheat streak mosaic virus	KS, USA	MK318276.1
KSWal2017	Wheat streak mosaic virus	KS, USA	MK318281.1
France_Marmagne	Wheat streak mosaic virus	France	HG810953.1
MON96	Wheat streak mosaic virus	MT, USA	AF511630.2
Naghadeh Iran	Wheat streak mosaic virus	Iran	EU914917.1
ONMV*	Oat necrotic mottle virus		NC005136.1
Sidney81	Wheat streak mosaic virus	NE, USA	AF057533.1
Sosn	Wheat streak mosaic virus	Poland	MH939146.1
Turkey1	Wheat streak mosaic virus	Turkey	AF454455.1
WA94	Wheat streak mosaic virus	WA, USA	FJ348358.1
WA99	Wheat streak mosaic virus	WA, USA	AF511643.2
WSMV_OH1	Wheat streak mosaic virus	OH, USA	MK975887.1
WSMV_TYPE	Wheat streak mosaic virus	KS, USA	AF285169.1
KSGre2017	Triticum mosaic virus	KS, USA	MK318272.1
COKCar	Triticum mosaic virus	CO, USA	MT762125.1
KSHm_2015	Triticum mosaic virus	KS, USA	MK318273.1
KSIct2017	Triticum mosaic virus	KS, USA	MK318274.1
NE	Triticum mosaic virus	NE, USA	FJ669487.1
U06-123	Triticum mosaic virus	KS, USA	FJ263671.1
YN-YZ211*	Sugarcane streak mosaic virus		KJ187047.1
CalVA KP1*	Caladenia virus A		JX156425.1

*These viruses were used as outgroups for the phylogenetic analysis

Supplementary Table 4. List of complete viral genome sequences and characterization of the consensus sequence of Triticum mosaic virus identified on wheat samples using Nanopore sequencing.

Sample ID	County	Accession number	No of reads†	Coverage (X)	Nucleotide identity (%)‡
19GT	Grant	OR900932	3228	109.63	99.7
19SW	Seward	OR900930	6891	316.79	99.7
19HM1	Hamilton	OR900929	7776	324.93	99.7
19MT	Morton	OR900931	3152	160.25	99.7
20GL2	Greeley	OR900928	6851	542.47	99.7
21WH1	Wichita	OR900923	3750	129.6	99.6
21WH2	Wichita	OR900924	14040	892.22	99.7
21WH3	Wichita	OR900925	3041	110.26	99.7
21LE3	Lane	OR900933	4939	213.8	99.7
21WH4	Wichita	OR900926	3881	126.13	99.7
21WH6	Wichita	OR900927	9071	391.06	99.6

† Number of reads obtained using nanopore sequencing and mapped with reference genome using CLC Genomics Workbench

‡Percent nucleotide identity of Kansas isolates sequence of this study to the reference genome TriMV KS isolate, FJ263671.1) sequence

Supplementary Table 5. List of complete viral genome sequences and characterization of the consensus sequences of High Plains wheat mosaic emaravirus identified on wheat samples using Nanopore sequencing.

Sample ID	county	Genome	Accession number	No of reads†	Coverage (X)‡	Nucleotide identity (%)‡
20SC2	Scott	RNA1	OR900934	1181	67.4	99.4
20SC2	Scott	RNA2	OR900935	1291	159.7	99.4
20SC2	Scott	RNA3A	OR900936	2982	912.5	99.6
20SC2	Scott	RNA3B	OR900940	67011	18320.7	98.3
20SC2	Scott	RNA4	OR900943	6970	650.0	99.1
20SC2	Scott	RNA5	OR900947	198	43.3	99.6
20SC2	Scott	RNA6	OR900949	869	194.8	99.4
20SC2	Scott	RNA7	OR900950	2232	757.0	99.1
20SC2	Scott	RNA8	OR900953	1222	37.7	98.8
20MC2	Mitchell	RNA3A	OR900937	370	16.1	96.3
20MC2	Mitchell	RNA3B	OR900941	972	250.6	99.3
20MC2	Mitchell	RNA4	OR900946	494	12.87	97.7
20KE2	Kearny	RNA3A	OR900938	350	97.0	98.1
20KE2	Kearny	RNA3B	OR900942	8470	1995.0	99.4
20KE2	Kearny	RNA4	OR900944	1716	650.0	98.6
20KE2	Kearny	RNA6	OR900948	121	16.0	99.1
20KE2	Kearny	RNA7	OR900951	369	88.6	98.3
20KE2	Kearny	RNA8	OR900954	280	10.0	98.8
21LE3	Lane	RNA3A	OR900939	634	157.4	98.7
20RH2	Rush*	RNA4	OR900945	1289	233.4	-
20RH2	Rush	RNA7	OR900952	655	90.6	85.5

† Number of reads obtained using nanopore sequencing and mapped with reference genome using CLC Genomics Workbench

‡Percent nucleotide identity of Kansas isolates sequence of this study to the reference genomes sequence

*Missing 115 bps at 5’ end and excluded from nucleotide percentage identity analysis only protein sequences were submitted to GeneBank

Supplementary Table 6. List of complete viral genome sequences and characterization of the consensus sequence of Soilborne wheat mosaic virus identified on wheat samples using Nanopore sequencing.

Sample ID	county	Accession numbers	Genome	No of reads†	Coverage (X)	Nucleotide identity (%)‡
19PN2	Pawnee	OR886214	RNA1	12818	816.55	98.47
19PN2	Pawnee	OR886216	RNA2	12723	596.12	98.52
21RL5	Riley	OR886215	RNA1	300	124.94	96.63
21RL5	Riley	OR886217	RNA2	1982	105.2	99.97

† Number of reads obtained using nanopore sequencing and mapped with reference genome using CLC Genomics Workbench

‡The reference genome used to compare the nucleotide identity were (RNA1: NC_002041.1 and RNA2: NC_002042.1)

Supplementary Table 7. List of sequences of High Plains wheat mosaic emaravirus (HPWMoV) isolates retired from GenBank.

Sample ID	RNA	virus	Origin	Accession Number
W1	RNA3	HPWMoV	OH, USA	KT970501.1
Cophil	RNA3	HPWMoV	CO, USA	MT762120.1
K1	RNA3	HPWMoV	OH, USA	KT98889.1
H1_RNA3	RNA3	HPWMoV	OH, USA	KT98881.1
NW2P3	RNA3	HPWMoV	OH, USA	MN250347.1
NE_RNA3A*	RNA3A	HPWMoV	NE, USA	KJ939625.1
KS7_3A	RNA3A	HPWMoV	KS, USA	KT988862.1
NW1_3A	RNA3A	HPWMoV	OH, USA	MN250339.1
NE_RNA3B*	RNA3B	HPWMoV	NE, USA	KJ939626.1
KS7_RNA3B	RNA3B	HPWMoV	KS, USA	KT988863.1
GG1_RNA3B	RNA3B	HPWMoV	OH, USA	KT988872.1
NE_RNA1*	RNA1	HPWMoV	NE, USA	NC_029570.1
NE_RNA2*	RNA2	HPWMoV	NE, USA	NC_029549.1
NE_RNA4*	RNA4	HPWMoV	NE, USA	NC_029551.1
NE_RNA5*	RNA5	HPWMoV	NE, USA	NC_029552.1
NE_RNA6*	RNA6	HPWMoV	NE, USA	NC_029553.1
NE_RNA7*	RNA7	HPWMoV	NE, USA	NC_029554.1
NE_RNA8*	RNA8	HPWMoV	NE, USA	NC_029555.1
RLBV**	RNA3	Raspberry leaf blotch virus	-	FR823301.1

*These HPWMoV isolates used as viral reference genomes used for mapping

**These viruses were used as outgroups for the phylogenetic analysis

Supplementary Table 8. Potential recombinant isolates of wheat streak mosaic virus (WSMV) analyzed by using 7 different algorithms in the RDP5 program

Recombinants	Major parent	Minor parent	RDP*	BootScan*	GENECONV*	MaxChi*	Chimera*	3seq*	SiScan*
19SV	Germany Hoym	WSMV type	1.51 x 10^{-36}	5.42 x 10^{-26}	1.85 x 10^{-24}	1.02 x 10^{-11}	3.25 x 10^{-12}	6.98 x 10^{-07}	4.47 x 10^{-13}
20GO	H95S	19RH1	4.37 x 10^{-48}	2.89 x 10^{-41}	3.6 x 10^{-39}	1.19 x 10^{-20}	3.57 x10^{-21}	2.84 x 10^{-37}	2.61 x 10^{-30}
20WA	20LE1	19RH1	6.16 x 10^{-63}	9.33 x 10^{-17}	4.92 x 10^{-12}	8.56 x 10^{-06}	4.33 x 10^{-06}	1.48x 10^{-09}	2.12 x 10^{-03}
19ST	19RH1	Sydney81	1.21 x 10^{-95}	-	1.38 x 10^{-04}	6.62 x 10^{-10}	2.28 x 10^{-06}	-	2.46 x 10^{-46}
19RA3	19SH3	19GH1	5.99 x 10^{-65}	1.46 x 10^{-03}	-	1.69 x 10^{-07}	1.06 x 10^{-05}	5.72 x 10^{-05}	3.43 x 10^{-11}
20EW	Sydney81	19SH3	3.13 x 10^{-22}	1.24 x 10^{-08}	2.16 x 10^{-07}	1.72 x 10^{-10}	2.04 x 10^{-08}	8.43 x 10^{-09}	6.52 x 10^{-11}
20TR2	19MT	19GH2	1.27 x 10^{-11}	3.42 x 10^{-04}	2.35 x 10^{-03}	4.28 x 10^{-06}	1.26 x 10^{-05}	2.02 x 10^{-03}	3.8 x 10^{-14}
20GH2	21RL1	21WH3	9.28 x 10^{-04}	2.79 x 10^{-03}	1.46 x 10^{-03}	1.93 x 10^{-03}	2.45 x 10^{-03}	3.29 x 10^{-04}	1.5 x 10^{-06}
20JW3	19DC1	20MC2	-	4.11 x 10^{-03}	-	1.6 x 10^{-03}	1.01 x 10^{-03}	9.61 x 10^{-04}	6.41 x 10^{-05}
19TR1	19CN1	20MC2	3.09 x 10^{-04}	-	-	2.22 x 10^{-03}	1.42x 10^{-03}	3.23x 10^{-07}	1.685x 10^{-03}
19FI	19GH1	19SH3	4.07 x 10^{-03}			1.82x 10^{-03}	1.42x 10^{-03}	3.23x 10^{-07}	1.685x 10^{-03}
19NS2	20SD4	MON96	1.28 x 10^{-08}	4.19 x 10^{-02}	-	3.37 x 10^{-05}	1.48 x 10^{-07}	1.8 x 10^{-12}	-
19GH1	21RL1	21WH3	5.97 x 10^{-06}	7.48 x 10^{-05}	2.29 x 10^{-03}	-	-	2.75 x 10^{-03}	1.32 x 10^{-03}
19SW	20MC2	19DC1		2.27 x 10^{-03}		5.44 x 10^{-06}	6.08 x 10^{-06}	2.62 x 10^{-06}	5.99 x 10^{-03}

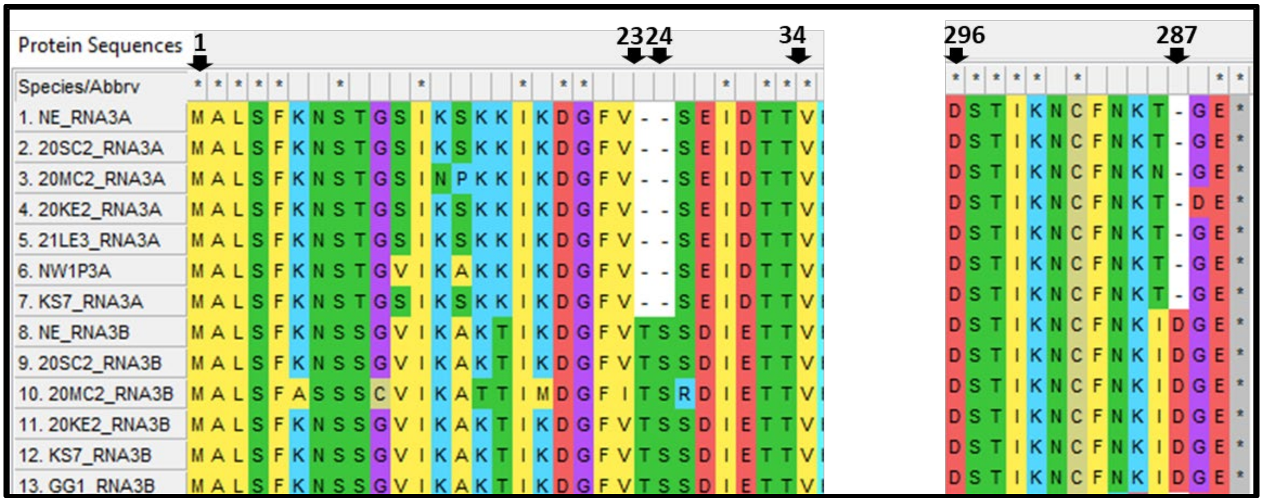

Supplementary Figure 1. Alignment of nucleocapsid protein sequence encoded by High Plains wheat mosaic emaravirus RNA3 and its two variant RNA3A and 3B isolates obtained from this study as well as isolates sequence retrieved from GenBank. RNA3B showed a 3 amino-acid insertion at the positions of 23, 24, and the 287 C terminus of the protein.
